# Telemedicine Breastfeeding Consultation: The Saudi Experience

**DOI:** 10.7759/cureus.45392

**Published:** 2023-09-17

**Authors:** Fouzia Abdulaziz AlHreashy, Gazi Ali AlObeid, Bushra M. A. Elbashir, Albandri Saleh Alshathry

**Affiliations:** 1 Family Medicine, General Directorate of Nutrition, Deputyship of Therapeutic Services, Ministry of Health, Riyadh, SAU; 2 Health Education, Al-Ahsa Health Directorate, Ministry of Health, Al-Ahsa, SAU; 3 Clinical Nutrition, General Directorate of Nutrition, Deputyship of Therapeutic Services, Ministry of Health, Riyadh, SAU

**Keywords:** telelactation, breastfeeding, satisfaction, counseling, consultation, telemedicine

## Abstract

Background

Telemedicine is widely used in health specialties. Yet, the experience of telemedicine use and its effectiveness in breastfeeding support is a research gap. The purpose of this study was to describe the pattern of telemedicine use for breastfeeding support in Saudi Arabia and to explore patients’ satisfaction with the service.

Methods

A cross-sectional survey was conducted in outpatient settings at Ministry of Health facilities in several Saudi regions. The number of breastfeeding consultations over one year (2021) was analyzed in terms of region, month, method of consultation, and women’s maternity status. Data on patient satisfaction and telemedicine techniques were gathered during 2022.

Results

Across the 16 regions enrolled in the project, 51,571 remote breastfeeding consultations were conducted, representing 28.2% of the total consultations. The eastern region reported the highest percentage (40.7%), and the southern region reported the lowest (2.4%). Almost two-thirds of the consultations were provided to lactating mothers (62.91%). Most data (90%) were collected from breastfeeding clinics in hospitals, and nurses were the main primary health care providers. The mean was 4,255 consultations per month. In terms of technology, telemedicine consultations were mostly conducted by phone (50%) and WhatsApp (38%). Satisfaction with telemedicine was reported by 80% of the participants, with a statistical difference found in those favoring telemedicine over in-person care (p=0.032), particularly for those using phone consultations and Telegram (p<0.001). WhatsApp respondents had the commonest neutral responses (p<0.001).

Conclusion

Telemedicine breastfeeding care has been widely established with high patient satisfaction. A national protocol outpatient lactation services with an intergrated hospital and primary care services and involvement of different health care professionals are recommended. Although breastfeeding counselling is proven to have a positive change on breastfeeding indicators, telemedicine tool per se needs further work on its role in breastfeeding indicators. Triage of cases to be evaluated face-to-face or referral to a specialist after telemedicine lactation care is an area for future work.

## Introduction

The coronavirus disease 2019 (COVID-19) pandemic caused unforeseeable global challenges in providing lactation training and skilled support, shortening postpartum stays, and preventing new mothers from receiving face-to-face breastfeeding support [1]. Fortunately, telemedicine emerged as a means to continue breastfeeding counseling remotely [2,3].

The World Health Organization (WHO) describes telemedicine as “The delivery of health care services, where distance is a critical factor, by all health care professionals using information and communication technologies for the exchange of valid information for diagnosis, treatment and prevention of disease and injuries, research and evaluation, and continuing education of health care providers, all in the interest of advancing the health of individuals and their communities” [4].

Since the COVID-19 pandemic began, telemedicine has been widely adopted throughout the world [5,6]. In Saudi Arabia, virtual care is a major initiative in the Saudi health care model [7] and one that was accelerated by the COVID-19 pandemic.

Breastfeeding consultation refers to comprehensive breastfeeding education, support, counseling, clinical management, and interventions given to women during the antenatal, perinatal, and postpartum periods to support the initiation and maintenance of breastfeeding, including those provided to women who experience difficulties breastfeeding due to anatomic variations, complications, and feeding problems with newborns. Distance counseling and/or other technologies may be very helpful in special settings, such as emergencies, where face-to-face counseling may be supplemented but not replaced by telephone counseling and/or other technologies [8]. Telehealth for breastfeeding consultation (i.e., telelactation) has existed for many years, mainly through phone consultation [9], and appears to be of particular importance in remote areas [10,11]. Despite an increase in the use of telehealth in the provision of breastfeeding support and education, the technology platforms were spotty [12].

Generally, patient satisfaction with virtual consultation has been reported to be high during COVID-19, although groups have perceived the impact of this new mode of clinical practice differently [1,10,13,14]. This variation in perception has also been reported regarding telelactation, with mothers with security concerns being six times more uncomfortable than mothers without such worries [15].

The effectiveness of using telemedicine for breastfeeding consultation is currently an area of academic interest. Gavine et al. determined the effectiveness of remote breastfeeding management to be low, highlighting the need for more research on this new technological tool [12]. Another study found telemedicine useful for communicating with other institutions and specialists and for seeking consultations on a variety of medical and surgical problems, psychological issues, medical reports, lab follow-ups, and medication refills, frequently noting that it is not a replacement for the physician’s hands-on expertise [16]. Systematic reviews of primary care in hospital settings have found lactation telehealth to be a promising method of care [17,18].

The evidence-based practice of using telemedicine for breastfeeding support is growing. For the purpose of reporting the Saudi experience of telemedicine consultation in breastfeeding, this study sought to describe the patterns of telemedicine in breastfeeding consultations at Saudi Arabian Ministry of Health (MOH) health care facilities and to measure clients’ satisfaction with remote breastfeeding counseling as compared to face-to-face consultation.

## Materials and methods

A descriptive cross-sectional study was conducted at MOH health care facilities (transforming to health clusters) during 2022. MOH health care facilities represent 60% of the total health care in Saudi Arabia [19]. Twenty administrative regions belong to MOH, each with a local breastfeeding coordinator. An operational project titled “Virtual Lactation Consultation” was set up in April 2020 to support MOH health care facilities; these facilities (hospital and primary care) were invited to engage in the project depending on the feasibility of manpower and technology support. The facilities were supported via a protocol for operating a breastfeeding clinic, training courses, the provision of virtual access (Anat platform ,i.e., a virtual application for video-based consultation linked to the Sehhaty appointment system), meetings to exchange experiences, and the establishment of key indicators for monitoring breastfeeding clinics and other types of breastfeeding services. For the purposes of this research, MOH health facilities were organized by geographical distribution into the five regions of Saudi Arabia (Central, Northern, Eastern, Western, and Southern). Four administrative regions were excluded from the study as a result of their lack of telehealth breastfeeding services or communication difficulties (one in the north and three in the south).

Pregnant women and breastfeeding mothers (representing the period of lactation) who had received outpatient breastfeeding consultations during 2021 were included in the study regardless of race or nationality; these included mothers who had been discharged from the hospital while their babies remained in the neonatal ward. Women who had received breastfeeding consultation services as inpatients were excluded from the study. Monthly indicators were collected from the involved health care facilities and entered into an Excel form containing the name of the region, health facility, and project coordinator in the region. The indicators (variables) included the month/year, number of lactation consultations with pregnant women (face-to-face or telemedicine), number of lactation consultations with breastfeeding mothers (face-to-face or telemedicine), and the number of lactation consultations (face-to-face) with a mother’s companion. Secondary resource data from outpatient breastfeeding consultation indicators for the year 2021 were utilized.

To gain a full picture of the patterns of telemedicine in Saudi Arabia, the Arabic language was used to explain the method of consultation and the satisfaction degree to the patients (Appendix). The survey was administered in Arabic via a Google link, by phone, or by SMS. A convenience sample from cases who consulted for breastfeeding was randomly collected from five geographical regions over the months of September and October 2022.

The operational project was supported by the deputyship of therapeutic services at MOH. The research was approved by the Institutional Review Board of MOH’s Studies and Research General Department (national registration number H-01-R-009; log number: 22-22 M). Permission was obtained from the data office at the MOH to utilize the project data for research purposes and from institutional research committees belonging to MOH directorates or health clusters from almost all the regions. Patient consent was obtained after consultation. Participation was optional, and the respondents’ data were kept anonymous.

The collected data were analyzed using Excel and Social Science Statistics online platform [20]. The number of consultations was computed using Excel. Descriptive statistics (frequencies and percentages) were used to describe the categorical data. A chi-square statistical test was used comparison of two variables. A p-value of ≤0.05 was used to report the statistical significance of the associated variables. The main outcome measures were the number of telemedicine consultations in breastfeeding and patient satisfaction using a 5-point Likert scale.

## Results

In total, 16 Saudi Arabia regions with MOH facilities joined the “virtual lactation consultation” project: Central (Riyadh and Qassim), East (Eastern area, Al-Ahsa, and Hafer Al Batin), North (Hail, Jouf, Tabuk, and Qaryat), West (Mecca, Jeddah, Taif, Al Medina, and Al Qunfudhah), and South (Asir and Najran). The response rate for the facilitation of the research and data sharing or collection was 90% across the regions.

Data collection revealed that telemedicine breastfeeding consultations were ongoing in both hospitals and primary care facilities; however, most data (90%) came from hospitals’ outpatient breastfeeding services. Hospital-based telemedicine was provided in antenatal care, health education, and breastfeeding clinics, or through a breastfeeding hotline service. Breastfeeding consultations through telemedicine in primary care were recorded in four regions (Al Ahsa, Riyadh First Health Cluster, Jouf, and Jeddah), where they were integrated with other services; that is to say, these areas lacked specialized breastfeeding clinics. In terms of the profession of the health care practitioners providing telemedicine breastfeeding consultation, the figures were as follows: nurses (66.7%), health educators (17.3%), physicians (12.8%), and dieticians and others (3.2%).

In total, 182,745 outpatient breastfeeding consultations were carried out in the participating regions in 2021. The highest number of consultations was reported in the Central region (36.3%), with the lowest in the Southern region (3.8%) of Saudi Arabia. The percentages of outpatient breastfeeding consultations provided were 62.5% and 37.5% for lactating women and pregnant women, respectively, as shown in Table 1. A data analysis found that breastfeeding consultations provided for women’s companions were undertaken in Al-Ahsa (Eastern region) (25.6%), Jeddah (Western region) (24.4%), and Najran (Southern region) (14.5%).

**Table 1:** Pattern of outpatient breastfeeding consultation in the regions of Saudi Arabia

	Method of Consultation				Total, N (%)
	Telemedicine		Face to Face		
	Pregnant, N (%)	Lactating, N (%)	Pregnant, N (%)	Lactating, N (%)	
Region					
Central	4,461 (23.3)	7,643 (23.6)	15,148 (30.7)	39,033 (47.7)	66,285 (36.3)
Eastern	4,734 (24.8)	13,199 (40.7)	12,213 (24.7)	22,349 (27.3)	52,495 (28.7)
Western	7,369 (38.5)	6,796 (21.0)	13,675 (27.7)	13,720 (16.8)	41,560 (22.7)
Northern	2,156 (11.7)	4,043 (12.5)	5,854 (11.9)	3,427 (4.2)	15,480 (8.5)
Southern	407 (2.1)	763 (2.4)	2,505 (5.1)	3,250 (4.0)	6,925 (3.8)
Total	19,127 (10.4)	32,444 (17.8)	49,395 (27.0)	81,779 (44.8)	182,745 (100)

**Figure 1:**
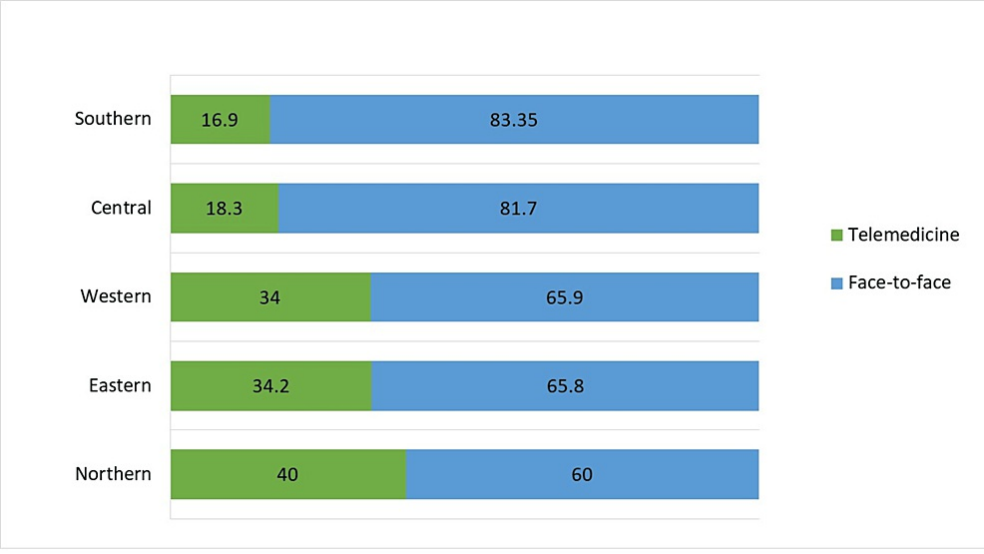
Method of outpatient breastfeeding consultation within each Saudi region

Research data from 2021 were subclassified within each region and showed that health care facilities in the Northern and Eastern regions of Saudi Arabia most utilized telemedicine in breastfeeding outpatient care at 40.0% and 34.2%, respectively, as shown in Figure 1.

**Figure 2:**
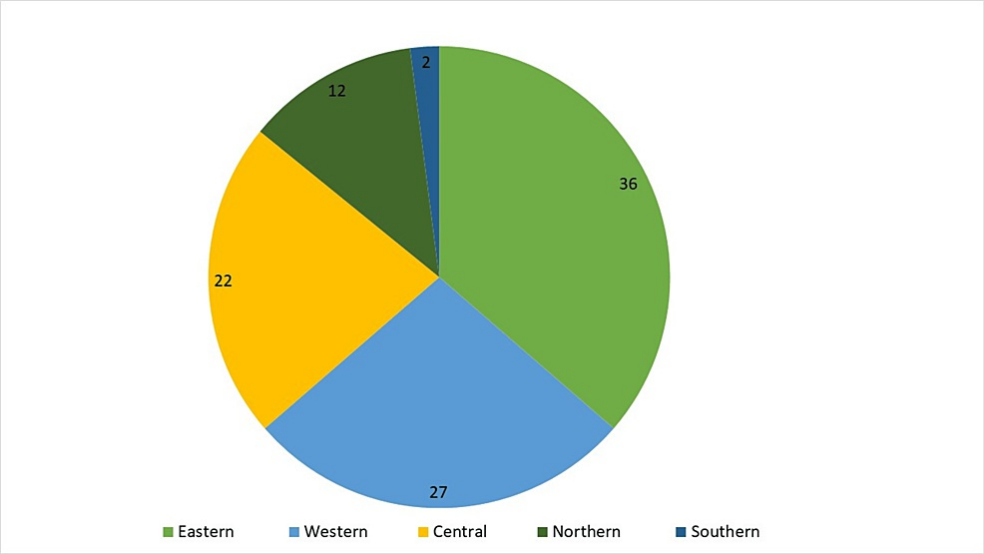
The percentage of telemedicine breastfeeding consultations per Saudi region

Furthermore, 51,571 telemedicine breastfeeding consultations were carried out in MOH facilities, representing 28.22% of total consultations, the highest number of which was reported in the Eastern region (40.7%) and the lowest (2.4%) in the Southern region, as shown in Figure 2. The most prevalent technology used for telelactation was the phone (50%) and WhatsApp (38 %), as shown in Table 2.

**Table 2:** Satisfaction according to the method and technology of telemedicine consultation

Patients’ Satisfaction	Method of Consultation			Telemedicine Type						
	Face to Face, N (%)	Telemedicine, N (%)	P-value	Telephone, N (%)	WhatsApp, N (%)	Video, N (%)	Telegram, N (%)	E-mail, N (%)	Instagram, N (%)	P-value
Very satisfied	2,859 (41.3%)	858 (43.5%)	0.072	561 (57.4%)	225 (29.9%)	43 (39.4%)	7 (9.1%)	22 (42.3%)	0	<0.001
Satisfied	2,641 (38.1%)	804 (40.8%)	0.032	334 (34.2%)	332 (44.1%)	58 (53.2%)	59 (76.6%)	20 (38.5%)	1 (50.0%)	<0.001
Neutral	1,089 (15.7%)	222 (11.3%)	<0.001	51 (5.2%)	153 (20.3%)	5 (4.6%)	5 (6.5%)	8 (15.4%)	0	<0.001
Unsatisfied	264 (3.8%)	71 (3.6%)	0.667	27 (2.8%)	33 (4.4%)	3 (2.8%)	5 (6.5%)	2 (3.8%)	1 (50.0%)	0.243
Very unsatisfied	68 (0.9%)	14 (0.7%)	0.267	4 (0.4%)	9 (1.2%)	0	1 (1.3%)	0	0	0.156
Total, N (%)	6,921 (77.9%)	1,969 (22.1%)		977 (11.0%)	752 (8.5%)	109 (1.2%)	77 (0.9%)	52 (0.6%)	2 (0.02%)	

**Figure 3:**
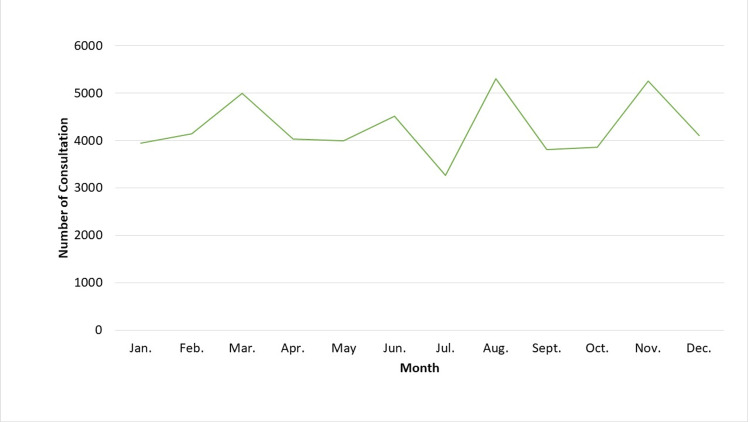
Monthly telemedicine breastfeeding consultations during 2021

The number of outpatient breastfeeding consultations carried out monthly in the facilities that engaged in the project during 2021 is illustrated in Figure 3. The number ranged between 3,270 and 5,307, and the mean was 4,255. Outpatient breastfeeding teleconsultations were carried out least frequently in July.

A total of 8,894 women responded to the satisfaction survey. Most women (more than 80%) were satisfied with their breastfeeding consultation regardless of the method used (face-to-face or via telemedicine); however, Table 2 shows that satisfaction was more statistically significant in the telemedicine group (40.8%) than in the face-to-face group (38.1%) (p=0.032), and there were more neutral responses in the face-to-face breastfeeding consultation group (p<0.001). Regarding satisfaction with the technology used in telemedicine, a statistically significant difference was observed (Table 2): phone consultation respondents were very satisfied, and Telegram respondents were satisfied (p<0.001). WhatsApp respondents showed significantly more neutral responses than those who had used other telemedicine methods (p<0.001). No statistical difference was observed between face-to-face and telemedicine respondents or within telemedicine methods among unsatisfied or very unsatisfied respondents, as shown in Table 2.

## Discussion

This study addressed the establishment of breastfeeding support in MOH facilities in outpatient settings and suggests development of breastfeeding management services and the enhancement of the virtual care experience among health care professionals. An increase in consultations in the Central, Eastern, and Western regions is proof that the “virtual care initiative” has helped improve breastfeeding health services at various levels of care. This could help slow the regions’ declining rates of exclusive breastfeeding [21-23] and may ultimately fulfill one of the WHO’s six global nutrition targets for 2025 [24].

However, it seems that outpatient breastfeeding consultations are run, to a large extent, in outpatient maternity hospitals. One of the factors driving this situation could be the breastfeeding counselors’ recommendation that mothers contact a hospital’s designated breastfeeding clinic following delivery, if necessary. Additionally, women may choose remote care over a hospital visit due to the medical safeguards put in place to protect against the spread of COVID-19. Other factors could include convenience, time savings, or prior positive experiences with telehealth. Virtual care is well established in primary care; however, the integrative approach may hinder the setup of specialized clinics and may be a barrier to the registry of breastfeeding cases. Primary care in Saudi Arabia is currently undergoing transformation, with an emphasis on preventive care [25]; this may prevent the establishment of specialized services in primary care, such as breastfeeding. The proportion of telemedicine consultations revealed in this research (28%) in relation to the total number of consultation reflects the project’s objective of strengthening continuous care of the counseling offered during telelactation rather than replacing face-to-face consultation [8].

 The nurse’s role in breastfeeding management is well documented [26]. It is suggested that Arabic-speaking nurses who are trained lactation consultants should be hired to expand the number of breastfeeding clinics. However, some medical situations call for the advice of qualified medical professionals, highlighting the importance of integrative teamwork [27]. Surprisingly, few dieticians were involved in breastfeeding consultations in this survey, which may be explained by the fact that dieticians have little exposure to maternal and child health, that nutrition clinics tend to use an integrated registry of cases, or that few dieticians work in MOH facilities [28].

Most MOH regions in Saudi Arabia use telemedicine, yet the proportion of cases served varied widely. The variability across regions correlated with population, number of maternity hospitals, availability of technology, existing breastfeeding services, and/or miscommunication issues with the region. According to the survey conducted for this study, Eastern areas have higher breastfeeding rates, and Al-Ahsa had more involvement of companions in breastfeeding counseling session [29,30]. Although the highest amount of breastfeeding was recorded to be in the southern area, this region participated least in this project. Exclusive breastfeeding is still not the primary choice made by women in the south, and there is a need to prioritize these areas to remove obstacles to breastfeeding [31]. Breastfeeding consultation services undoubtedly continued during 2021 and 2022, but July was a summer break period and coincided with Hajj (pilgrim) season, during which outpatient services are significantly scaled back. The national holiday may be behind the gap in clinical care in infant nutrition.

Phone consultation is the oldest method and the most utilized technology in telemedicine [9], which may account for its availability among both providers and clients. WhatsApp is the most popular application among Saudis for general social networking; hence, it was commonly mentioned by the studied population. However, it also had the most neutral satisfaction ratings from survey respondents, suggesting the importance of an interactive method of communication in telemedicine. Interestingly, a systematic review found that chat-based support care is effective in high-income communities [32,33], and this may positively speculate an effect of WhatsApp use in this survey. Despite the availability of virtual (video)-based care via the Anat application in some regions, its utilization remains low. Seguranyes et al. found that videoconferencing and telephone contact were more effective tools than face-to-face standard care of mothers attending health centers in reducing the number of health center visits, and that these methods allow mothers to consult health staff immediately and from their own homes [34]. Overall, this study found patient satisfaction with telemedicine to be high in the Saudi population, as reported in other studies [1,13,35].

The study was limited in terms of knowledge about the content of breastfeeding cases, the triaging pathway for in-person consultations, the description of antenatal education in breastfeeding, and the reason behind limited videoconferencing. Additionally, the sample size was not calculated in advance of the study. Other health care facilities in Saudia Arabia, which belongs to private or other governmental sectors, were not included in this study, hindering the full representation of the country. The response rate for satisfaction rate could not be calculated in this survey. When this study is added to the existing literature, future research should consider the efficacy of telemedicine used in the studied population [36,37].

## Conclusions

This study on the use of virtual lactation consultation was an opportunity to introduce technology into the health care of new mothers, potentially enhancing the experiences of both health care providers and patients. To ensure equity of care in outpatient breastfeeding support, both face-to-face and virtual care are necessary. This involves the integration of primary and hospital care in breastfeeding support. Team work of different health care professionals such as physicians and dieticians is necessary to empower the lactation services, and a national protocol for outpatient breastfeeding consultation is advisable. Establishment of a breastfeeding hotline during Hajj season is recommended. The authors encourage future examinations of the effectiveness of such services in each region, particularly in relation to breastfeeding practice indicators.

## Appendices

Dear pregnant lady and lactating mothers,

Thank you for your consultation on breastfeeding, Your response to these two questions is valuable for research purposes to improve health services. It is voluntary to respond to it.

 What was the method you have used for breastfeeding consultation (you can choose more than one:

□        Phone

□        Video (Sehhaty and Anat)

□        WhatsApp message

□        Telegram

□        Email

□        Zoom

□        Microsoft teams

□        Face to face (on site)

□        Other

What is your satisfaction with the distance breastfeeding consultation done with you?

□        Very satisfied

□        Satisfied

□        Neutral

□        Unsatisfied

□        Very unsatisfied  

## References

[1] Abdel Nasser A, Mohammed Alzahrani R, Aziz Fellah C, Muwafak Jreash D, Talea A Almuwallad N, Salem A Bakulka D, Abdel Ra'oof Abed R (2021). Measuring the patients' satisfaction about telemedicine used in Saudi Arabia during COVID-19 pandemic. Cureus.

[2] Brown A, Shenker N (2021). Experiences of breastfeeding during COVID-19: Lessons for future practical and emotional support. Matern Child Nutr.

[3] Salvatore CM, Han JY, Acker KP (2020). Neonatal management and outcomes during the COVID-19 pandemic: an observation cohort study. Lancet Child Adolesc Health.

[4] (2023). Telemedicine: opportunities and developments in Member States: report on the second global survey on eHealth. https://apps.who.int/iris/handle/10665/44497.

[5] Bokolo Anthony Jnr (2020). Use of telemedicine and virtual care for remote treatment in response to COVID-19 pandemic. J Med Syst.

[6] Almathami HK, Win KT, Vlahu-Gjorgievska E (2020). Barriers and facilitators that influence telemedicine-based, real-time, online consultation at patients' homes: systematic literature review. J Med Internet Res.

[7] (2023). Health Sector: Transformation Strategy. Vision 2030. https://www.moh.gov.sa/en/Ministry/vro/Documents/Healthcare-Transformation-Strategy.pdf.

[8] (2023). Implementation Guidance on Counselling Women to Improve Breastfeeding Practices. https://www.globalbreastfeedingcollective.org/media/1501/file/UNICEF-WHO-BF-Counseling-Guidance-2021.pdf.

[9] Ferraz Dos Santos L, Borges RF, de Azambuja DA (2020). Telehealth and breastfeeding: an integrative review. Telemed J E Health.

[10] Uscher-Pines L, Ghosh-Dastidar B, Bogen DL, Ray KN, Demirci JR, Mehrotra A, Kapinos KA (2020). Feasibility and effectiveness of telelactation among rural breastfeeding women. Acad Pediatr.

[11] Grubesic TH, Durbin KM (2020). The complex geographies of telelactation and access to community breastfeeding support in the state of Ohio. PLoS One.

[12] Gavine A, Marshall J, Buchanan P (2022). Remote provision of breastfeeding support and education: systematic review and meta-analysis. Matern Child Nutr.

[13] Alharbi KG, Aldosari MN, Alhassan AM, Alshallal KA, Altamimi AM, Altulaihi BA (2021). Patient satisfaction with virtual clinic during Coronavirus disease (COVID-19) pandemic in primary healthcare, Riyadh, Saudi Arabia. J Family Community Med.

[14] Garcia-Huidobro D, Rivera S, Valderrama Chang S, Bravo P, Capurro D (2020). System-wide accelerated implementation of telemedicine in response to COVID-19: mixed methods evaluation. J Med Internet Res.

[15] Louis-Jacques AF, Schafer EJ, Livingston TA, Logan RG, Marhefka SL (2021). Modesty and security: attributes associated with comfort and willingness to engage in telelactation. Children (Basel).

[16] Mubaraki AA, Alrabie AD, Sibyani AK, Aljuaid RS, Bajaber AS, Mubaraki MA (2021). Advantages and disadvantages of telemedicine during the COVID-19 pandemic era among physicians in Taif, Saudi Arabia. Saudi Med J.

[17] Li Y, Dai J, Cui L (2020). The impact of digital technologies on economic and environmental performance in the context of industry 4.0: a moderated mediation model. Int J Prod Econ.

[18] Hubschman-Shahar LE (2022). Lactation telehealth in primary care: a systematic review. Breastfeed Med.

[19] Ministry of Health (2021). Ministry of Health Statistical Book.

[20] (2023). Social Science Statistics. https://www.socscistatistics.com/.

[21] Alyousefi NA (2021). Determinants of successful exclusive breastfeeding for Saudi mothers: social acceptance is a unique predictor. Int J Environ Res Public Health.

[22] Raheel H, Tharkar S (2018). Why mothers are not exclusively breast feeding their babies till 6 months of age? Knowledge and practices data from two large cities of the Kingdom of Saudi Arabia. Sudan J Paediatr.

[23] Alsulaimani NA (2019). Exclusive breastfeeding among Saudi mothers: Exposing the substantial gap between knowledge and practice. J Family Med Prim Care.

[24] (2023). Guideline: Counseling of women to improve breastfeeding practice. https://apps.who.int/iris/bitstream/handle/10665/280133/9789241550468-eng.pdf.

[25] Al Saffer Q, Al-Ghaith T, Alshehri A (2021). The capacity of primary health care facilities in Saudi Arabia: infrastructure, services, drug availability, and human resources. BMC Health Serv Res.

[26] (2023). Breastfeeding Best Practice Guidelines for Nurses. https://rnao.ca/sites/rnao-ca/files/Breastfeeding_Best_Practice_Guidelines_for_Nurses.pdf.

[27] Wilbur K, Snyder C, Essary AC, Reddy S, Will KK, Mary Saxon S (2020). Developing workforce diversity in the health professions: a social justice perspective. Health Prof Educ.

[28] (2023). Registered Dietitian Nutritionist Confidence and Competence to Promote and Support Breastfeeding: A Report on Survey Findings. https://cdr.lib.unc.edu/concern/masters_papers/dz010s01h.

[29] Al-Katufi BA, Al-Shikh MH, Al-Hamad RF, Al-Hajri A, Al-Hejji A (2020). Barriers in continuing exclusive breastfeeding among working mothers in primary health care in the ministry of health in Al-Ahsa region, Saudi Arabia. J Family Med Prim Care.

[30] Ahmed AE, Salih OA (2019). Determinants of the early initiation of breastfeeding in the Kingdom of Saudi Arabia. Int Breastfeed J.

[31] Mirghani E, Alghamdi JM, Ibrahim MM (2020). A survey on knowledge, attitude, and practice of female teachers about breastfeeding in Albaha Region KSA. Int J Health Sci Res.

[32] Almohanna AA, Win KT, Meedya S (2020). Effectiveness of internet-based electronic technology interventions on breastfeeding outcomes: systematic review. J Med Internet Res.

[33] Brody C, Star A, Tran J (2020). Chat-based hotlines for health promotion: a systematic review. Mhealth.

[34] Seguranyes G, Costa D, Fuentelsaz-Gallego C (2014). Efficacy of a videoconferencing intervention compared with standard postnatal care at primary care health centres in Catalonia. Midwifery.

[35] Wali R, Alhakami A, Alsafari N (2022). Evaluating the level of patient satisfaction with telehealth antenatal care during the COVID-19 pandemic at King Abdul-Aziz Medical City, Primary Health Care Center, Specialized Polyclinic. Womens Health (Lond).

[36] Naaz S, Asghar A (2022). Artificial intelligence, nano-technology and genomic medicine: the future of anaesthesia. J Anaesthesiol Clin Pharmacol.

[37] Marcucci B (2018). Use of telehealth to increase breastfeeding exclusivity and duration. Clin Lactation.

